# Active learning strategies for the design of sustainable alloys

**DOI:** 10.1098/rsta.2023.0242

**Published:** 2024-11-04

**Authors:** Ziyuan Rao, Anurag Bajpai, Hongbin Zhang

**Affiliations:** ^1^ Max Planck Institute for Sustainable Materials, Düsseldorf, Germany; ^2^ National Engineering Research Center of Light Alloy Net Forming School of Materials Science and Engineering, Shanghai Jiao Tong University, Shanghai, People‘s Republic of China; ^3^ Institut für Materialwissenschaft, Technische Universität Darmstadt, Darmstadt, Germany

**Keywords:** active learning, exploitation and exploration strategies, sustainable alloys, single-objective optimization, multi-objective optimization

## Abstract

Active learning comprises machine learning-based approaches that integrate surrogate model inference, exploitation and exploration strategies with active experimental feedback into a closed-loop framework. This approach aims at describing and predicting specific material properties, without requiring lengthy, expensive or repetitive experiments. Recently, active learning has shown potential as an approach for the design of sustainable materials, such as scrap-compatible alloys, and for enhancing the longevity of metallic materials. However, in-depth investigations into suited best-practice strategies of active learning for sustainable materials science are still scarce. This study aims to present and discuss active learning strategies for developing and improving sustainable alloys, addressing single-objective and multi-objective learning and modelling scenarios. As model cases, we discuss active learning strategies for optimizing Invar and magnetic alloys, representing single-objective scenarios, and more general steel design approaches, exemplifying multi-objective optimization. We discuss the significance of finding the right balance between exploitation and exploration strategies in active learning and suggest strategies to reduce the number of iterations across diverse scenarios. This kind of research aims to find metrics for a more effective application of active learning and is used here to advance the field of sustainable alloy design.

This article is part of the discussion meeting issue ‘Sustainable metals: science and systems’.

## 1. Introduction

Designing alloys with novel sets of physical properties is critical in enabling sustainable solutions in energy conversion, transport, households and industry [1,2]. However, the significant environmental impact linked to metal extraction and alloy synthesis necessitates not only that the metal components themselves have to contribute to sustainable technologies, but that their production processes must also transition to become more environmentally benign [3–5]. For example, designing metallic alloys from scrap is an essential approach to sustainable materials development, contributing to resource conservation and lower environmental impact [6,7]. For example, recycling aluminium from scrap rather than extracting it from ores provides substantial energy savings. The energy required to produce one single tonne of primary aluminium (referred to as primary production) from about 4–5 tonnes of bauxite ore is around 170–220 GJ. In contrast, producing aluminium from recycled materials (called secondary production) requires only about 5% of the energy compared to primary production [8–10]. Producing 1 tonne of steel from about 2 tonnes of hematite ore consumes about 20 GJ of energy per tonne. When instead produced from scrap through electric arc furnaces, the energy required is about 25–30% of this value [11–14].

Another approach for enhancing the sustainability of metals is to improve their resistance to environmental degradation because we do not need to replenish what does not have to be scrapped. Thus, enhancing the corrosion resistance of alloys ensures longevity and reliability of structural parts in multiple load-bearing applications while omitting the environmental impact associated with material degradation and replacement. However, designing materials with tailored properties—be it for high scrap tolerance or improved corrosion resistance—is a very complex and multi-faceted task. Conventional alloy development of such immense complexity has traditionally been done by applying thermodynamically and kinetically guided design rules in conjunction with often laborious and costly trial-and-error experiments needed to establish the resulting relations between chemical composition, structure, processing and properties. This traditional approach is time-consuming and often inefficient. Moreover, empirically developed material design rules can fail when the ever-increasing chemical complexity of modern advanced materials comes into play, such as for advanced steels [15,16], high-strength aluminium alloys, high-entropy alloys (HEAs) [17] or medium-entropy alloys (MEAs) [18,19].

Using machine learning (ML) to design materials under such multi-parameter constraints is an emergent field of materials research, and this trend is rapidly growing [20,21]. ML can accelerate material discovery due to the innovation of ML algorithms, more available information resources and databases and new promising automatable workflow connections between ML, large language models and classic simulation [22–24]. A typical example is the inverse design [25], which benefits from the rapid development of generative algorithms, e.g. variational autoencoder (VAE) [22,26] and generative adversarial network methods [23]. Another strong impetus comes from the rapid growth of databases, e.g. the AFLOW [27], OQMD [24], Materials Project [28] and NOMAD [29] repositories, which contain millions of calculations with hundreds of millions of properties [20]. In contrast, there are worldwide efforts to curate reliable experimental databases, as exemplified by the national research data infrastructure recently launched in Germany and the European materials modelling ontology initiative [30,31]. These large-scale efforts further motivate a closed-loop materials design framework, integrating ML modelling, theoretical simulations and experimental validations.

In comparison with rather linear and single feature-oriented property predictions in most previous ML-enabled materials design approaches [32,33], active learning has been proposed and applied as a next step in sustainable alloy design in recent years [34–36]. The term active learning refers in general to an ML technique where a model iteratively selects the most informative data from an unlabelled pool for labelling by an expert (referred to as an oracle). This approach enhances model efficiency by prioritizing data that the model finds either uncertain or valuable. The process starts by initializing with a small, well-labelled dataset, using the model to identify key unlabelled instances, having an expert label these data and then feeding back to the original dataset and updating the model accordingly. This iteration can be repeated sequentially, depending on data availability, until the defined performance target is reached or data resources are depleted.

So far, active learning has demonstrated its effectiveness in small datasets within materials science. For example, Xue *et al*. discovered low thermal hysteresis NiTi-based shape memory alloys Ti_50.0_Ni_46.7_Cu_0.8_Fe_2.3_Pd_0.2_, by synthesizing and characterizing 36 predicted compositions after 9 iterations based on a small dataset with only 22 original data [34]. Similar design processes have been applied to magnetocaloric materials [37], superconductors [37] and HEAs [38], among others. However, most such studies on active learning have so far mainly pursued the discovery of new types of structures and materials based on different datasets. Yet, detailed investigations into more holistic strategies to solve complex and multi-property and multi-constraint material problems are still scarce. In particular, more practical strategies are needed because iterating over all the suggested possibilities can be time- and resource-consuming. Although it is less pertinent to compare different material discovery processes across diverse scenarios, it makes sense to elaborate on a few more universal strategy approaches.

In this work, we focus on the study of active learning-based alloy design strategies for the development of Invar alloys, magnetic materials and steels using datasets curated from the literature [39–44]. Invar alloys are a type of material characterized by their exceptionally low thermal expansion coefficient (TEC) [45], making them suitable for emerging markets involving the transportation of liquid hydrogen, ammonia and natural gas. The magnetic material data we collected encompass both soft and hard magnetic alloys, which are important materials in energy conversion and utilization. The third group considered here, viz., Transformation-Induced Plasticity (TRIP) steels are high-strength ductile alloys for demanding sheet forming applications. Focusing here on the optimization of these alloys, we aim to illustrate the fundamental concepts and provide generic solutions for both single- and multi-objective optimization of sustainable alloys.

In the context of single-objective optimization, our initial step involves utilizing the autoencoder to visualize the whole data distribution from the corpora in latent space. The autoencoder approach harnesses a neural network (NN) framework, consisting of an encoder and decoder, for efficient dimensionality reduction. By gaining insights into the data distribution through visualization, we can subsequently identify inherent clusters and patterns. This approach is essential for two reasons. First, it becomes in principle visible if the underlying data are at all rich and detailed enough to reveal groups and patterns. In other words, these first steps help to explore if an ML approach makes sense in that context or not. Second, data clustering and certain shape metrics of such data clusters can act as a guide to identify and pursue pertinent material development directions.

**Figure 1.**
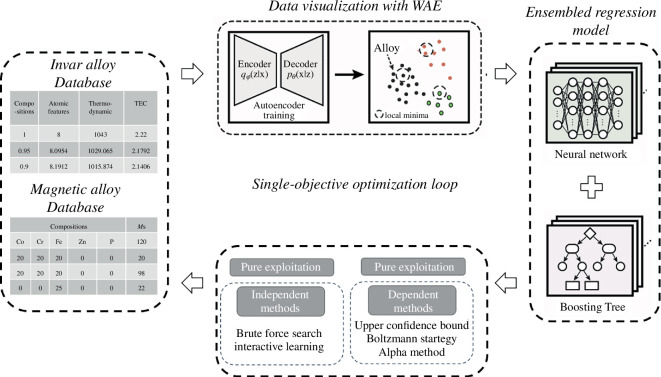
Overview of active learning framework for the single-objective optimization in this work. First, the alloys in the dataset are visualized with the Wasserstein autoencoder (WAE). Second, the alloys in the training dataset are trained with ensemble models comprised of neural networks and boosting trees. Third, different exploitation and exploration strategies are alternatively used to search for promising compositions in the test dataset. In the last step, the most promising compositions are selected and then fed back into the training dataset to initiate the next iteration. This figure is adapted from figure 1 in [50]. This framework is used to test the efficiency of the active learning strategies including pure exploitation, pure exploration, dependent and independent methods, respectively, for different dataset. The pure exploitation strategy prioritizes compositions with target mean values, such as those with low TEC for Invar alloys. Conversely, the pure exploration strategy seeks compositions with high uncertainty values to explore unknown regions. Independent methods conduct either exploration or exploitation in each iteration, while dependent methods combine both in each iteration. Further details of these methods are provided in Methods §2d.

In the subsequent section, following the division of the dataset into training and testing datasets, we then show how to train a model using the training dataset. To evaluate the efficiency of various active learning strategies, we sample promising alloy compositions from the test dataset, alternatively employing diverse approaches which are commonly used in previous publications [34,46–49] such as pure exploitation, pure exploration, dependent and independent methods with the exploitation–exploration combination. The pure exploitation strategy prioritizes new compositions with target mean values, like low TEC for Invar alloys, while the exploration strategy seeks compositions with high uncertainty values. Dependent and independent methods aim to balance the exploitation and exploration strategies. In the independent strategies, only one exploration or exploitation is conducted per iteration, whereas in the dependent methods, both are used per iteration. Further details are provided in §2d.4 and illustrated in figure 1. Our goal is to assess the efficiency of these strategies across different alloy datasets, specifically measuring how quickly they can identify compositions with desired properties (e.g. low TEC for Invar alloys and high magnetization for magnetic alloys) from the test dataset, in terms of required active learning iterations. A similar workflow is presented for multi-objective optimization of TRIP steels in electronic supplementary material, figure S1.

Through our comparison of various active learning strategies, we elucidated the significance of both exploitation and exploration strategies. Exploitation aids in identifying the optimal optimization path based on the current known probability distribution, while exploration actively explores an unknown space while preventing getting trapped in local optima. Furthermore, we delve into the intricacies of balancing these strategies to reduce the number of iterations required. In the last part, similar strategies are discussed for the multi-objective optimization active learning of TRIP steels, focusing on various ways to balance exploitation and exploration in such scenarios.

## 2. Methods

### (a) Database

Our Invar dataset has been curated from previous publications [38–44]. The alloys in the dataset include 717 different alloys, from simple binary alloys to complex MEAs and HEAs. The chemical compositions, atomic properties, density functional theory and thermodynamic calculations are used as input (i.e. descriptors) and TEC at room temperature as output. The magnetic alloy dataset comprises 246 distinct magnetic alloys, with chemical compositions serving as the sole input. The corresponding output is the saturation magnetization (M_S_). For the TRIP steel dataset, chemical compositions along with elemental attributes that affect the mechanical properties are considered as input. The details of the features and bias of the data are shown in electronic supplementary material, tables S1, S2 and figure S7.

### (i) Data distribution in the latent space

The Wasserstein autoencoder (WAE) was employed to visualize alloy distributions in latent space. As a variant of the VAE family [22], WAE is a constrained architecture that optimizes a loss function combining reconstruction and regularization terms, expressed as:


$$L=L_{\text{rec}}+L_{\text{reg}}.$$

In this study, binary cross-entropy was used for *L*_rec_, and the maximum mean discrepancy between the latent space distribution and a Gaussian prior for *L*_reg_ [51]. The WAE’s encoder and decoder each comprise three neural layers with 80, 64 and 48 nodes, respectively, using a leaky ReLU activation function with a slope of −0.02. Hyper-parameters were optimized via Bayesian optimization (BO), which efficiently searches complex spaces by balancing exploration and exploitation. The architecture was kept simple to prevent overfitting, focusing on tuning the number of epochs, batch size and learning rate. The training history is depicted in electronic supplementary material, figure S2.

### (b) Ensemble regression model and uncertainty calculation

The ensemble regression model is an ML approach that combines multiple individual regression models to improve predictive accuracy and robustness. Employing an ensemble regression model as a surrogate model in active learning is crucial because it offers not only the predicted mean value but also uncertainty estimation. The predicted mean value guides exploitation strategies, while uncertainty estimation informs exploration strategies.

The Invar and magnetic datasets were modelled using an ensemble approach, consisting of 10 NN models and 10 gradient-boosted decision tree (GBDT) models. NNs are basic ML models made up of layers of interconnected neurons, each layer transforming the input data to generate outputs. GBDT models enhance predictive accuracy by iteratively combining multiple decision trees, with each new tree correcting errors from the previous ones, resulting in a strong ensemble. The training and testing outcomes for the NN and GBDT models are displayed in electronic supplementary material, figures S3 and S4, respectively.

In addressing the TRIP steel dataset, training was conducted employing two discrete random forest (RF) regressors, one for yield strength and another one for ductility. RF is a versatile ML model that belongs to the ensemble learning family. It operates by constructing a multitude of decision trees during training and outputs the mode of the classes (classification) or the mean prediction (regression) of the individual trees. A total of 10 distinct RF models were deployed for training, each using a varied random training dataset. Subsequently, aggregation of model predictions was used to compute mean prediction values and related uncertainties. The optimal model parameters were determined through 10 000 iterations of random search followed by 10 iterations of Bayesian search, focusing on optimizing hyper-parameters such as the number of layers and nodes per layer. The mean absolute percentage error (MAPE) was selected as the loss function, which yields:


$$\text{MAPE}=\frac{100}{n}\Sigma_{i=1}^{n}\left|\frac{A_{i}-P_{i}}{A_{i}}\right|,$$

where $A_{i}$ is the actual value, $P_{i}$ is the prediction and *n* is the number of datapoints. The surrogate models trained on the TRIP steel datasets were evaluated using the *R*^2^ score and mean-squared error (m.s.e) values.


$$R^{2}=1-\frac{\sum {\left(A_{i}-P_{i}\right)}^{2}}{\sum {\left(A_{i}-\overset{-}{A}\right)}^{2}}$$

$$\text{MSE}=\;\frac{1}{n}\sum {\left(A_{i}-\overset{-}{A}\right)}^{2},$$

where $\overset{-}{A}$ is the average value, $A_{i}$ is the actual value, $P_{i}$ is the prediction and *n* is the number of the data.

### (c) Exploration and exploitation strategies for single-objective optimization

#### (i) Pure exploitation and exploration

The pure exploitation strategy prioritizes compositions with target mean values, such as those with low TEC for Invar alloys. Conversely, the pure exploration strategy seeks compositions with high uncertainty values to explore unknown regions.

We also employ various strategies to balance exploitation and exploration for single-objective optimization. These strategies fall into two categories: independent and dependent. Independent methods conduct either exploration or exploitation in each iteration, while dependent methods combine both in each iteration.

#### (ii) Dependent methods

The dependent methods are commonly used in previous publications [34,46–50] to balance exploitation and exploration for single-objective optimization. These methods provide a good compromise between exploration and exploitation strategies in each iteration, allowing the screened compositions to have the promise of becoming good performance alloys while simultaneously exploring the entire composition universe. We choose several mostly used dependent methods to be involved in this article and alternatively test their performance:

*Upper confidence bound (UCB)*: In this strategy, we select the alloys with the highest upper bound:


$$x=\arg\max_{x\;\in ℵ}\;\left({\tilde{\mu }}_{x}+\;\sigma_{x}\right),$$

where x is the most promising alloy composition, ${\tilde{\mu }}_{x}$ is the mean and $\sigma_{x}$ is the uncertainty. ℵ represents the whole composition space.

*Boltzmann strategy (BS)*: In this strategy, we first define a sampling probability using a softmax function of a Boltzmann type according to


$$P^{n}\left(x\right)=\;\;\frac{e^{\rho {\tilde{u}}_{x}^{n}}}{\sum_{x^{´}\in ℵ}e^{\rho {\tilde{u}}_{x^{´}}^{n}}},$$

where ${\tilde{\mu }}_{x}$ is the predicted mean and *ρ* is a constant. By gradually decreasing the *ρ*, we encourage exploration initially, developing towards exploitation over time. And on the contrary, by gradually increasing the *ρ*, we gradually increase the degree of exploration. Here, we use *ρ* = 1 to increase the degree of exploration.

*Alpha strategy*: We employ a rank-based objective function as a guide for our experiments [38,52], which mitigates the impact of outliers and eliminates inhomogeneity or skewness in the predicted TEC distribution by treating all ranks as equally spaced from each other:


Rankscore=α×rank_of_prediction+(1−α)×rank_of_uncertainty,

where rank_of_prediction is the rank of the predicted mean and rank_of_uncertainty is the rank of the predicted uncertainty. α is the weight to combine these two ranks. We choose the composition with the highest rank score as the most promising composition. By gradually increasing the weight, we gradually increase the degree of exploitation. Here, we use α = 0.8.

*BO:* We implement a Gaussian Process Regression (GPR)-based BO framework to guide the exploration of alloy compositions. The acquisition function, specifically the Expected Improvement (EI) criterion, balances exploration and exploitation by prioritizing compositions that offer the potential for the greatest improvement over the current best result. The EI is calculated as follows:


$$\text{EI}\left(X\right)\;=\;\left(\mu \left(X\right)\;-\;\mu_{best}\right)=\Phi \left(Z\right)+\sigma (x)\emptyset \left(Z\right),$$

where μ(x) is the predicted mean, σ(x) is the predicted uncertainty, $\mu_{best}$ is the current best mean, Φ(Z) is the cumulative distribution function and ∅(Z) is the probability density function of a standard normal distribution. The most promising composition is selected based on the maximum EI value. By fine-tuning the hyper-parameters of the GPR model, we optimize the trade-off between exploration of new alloy spaces and exploitation of known high-performing regions, thereby efficiently navigating the vast compositional design space.

#### (iii) Independent methods

As a compromise approach, dependent methods tend to overlook certain compositions with the highest uncertainties or compositions with the most promising properties. This limitation arises because dependent methods cannot simultaneously explore and exploit at 100% capacity in each iteration. To address this limitation, we introduce a complementary method, referred to as independent methods. Unlike dependent methods, which seek to balance exploration and exploitation within each iteration, independent methods balance these aspects across the entire process. Consequently, independent methods enable full exploration and exploitation in each iteration while also integrating both exploration and exploitation. The independent methods employed in this study include the following:

*Brute force search*: Brute force search means adding exploration at the initial iterations to explore the unknown regions and then using exploitation to discover alloys in the later iterations. Specifically, we first perform different times of exploration, namely 0, 2, 4, 5, 6, 7 and 8 explorations, and then use exploitation.

*Interactive learning*: Interactive learning involves initially prioritizing exploitation in the first few iterations. If exploitation yields no further gains, we then transition to exploration before returning to exploitation again.

### (d) Active learning for multi-objective optimization

The notion of the Pareto front (PF), which outlines the trade-offs involved in simultaneously improving many objectives, is central to multi-objective optimization. As shown in electronic supplementary material, figure S8, the axes of the PF plot represent the different properties, and a characteristic boundary is defined. This boundary includes materials where none of the objectives can be enhanced without compromising the value of the other objective. The non-dominated data points, known as boundary points, establish a PF, which signifies the optimal balance between the objectives [53]. The design aims to determine the unidentified PF using the available initial data while minimizing the need for additional measurements. After several iterations, the sub-optimal PF will transform into the optimal PF. Several techniques relating to such adaptive multi-objective design problems have been discussed in the literature [34,48,49] to maximize the EI, while selecting the next candidate data point, with a focus on minimizing the number of iterations required [54].

Electronic supplementary material, figure S8 represents the distribution of the initial training dataset along with the sub-optimal PF. The beige-shaded zone represents the area where a candidate alloy, after measurement, would lead to an enhancement compared to the current front indicated by the purple dots. This implies that the existing sub-optimal PF would be altered to incorporate the newly measured alloys. The probability of improvement *P(I)* that the new point is better than all existing points is the cumulative probability of a candidate data point integrated over the beige-shaded region.


$$P(I)=\int \varphi \left(y_{1},y_{2}\right)dy_{1}dy_{2}.$$

The variables $y_{1}$ and $y_{2}$ are the targets and the function $\varphi (y_{1},y_{2})$ is a Gaussian probability distribution function that is estimated by combining the mean and variance of the $y_{1}$ and $y_{2}$ distributions. This function may be expressed as $\varphi (y_{1},y_{2})$ = $\varphi (y_{1})\varphi (y_{2})$, indicating that it is uncorrelated. Consequently, we have made the assumption that the anticipated values follow a Gaussian distribution characterized by a mean and variance. Similarly, the two objective EI is identical. The expression *E[I(x)]* is the first moment of the function *I* applied to the joint probability distribution $\varphi (y_{1},y_{2})$ over the beige area shown in electronic supplementary material, figure S8 concerning the current sub-optimal PF.

From a geometric perspective, the calculation of *E[I(x)]* yields the value of *P[I(x)]L.* The value of *L* can be evaluated in two different ways to balance exploration and exploitation: either using the Centroid approach or the Maximin approach.

The EI-Centroid [55] methodology is a method of calculating the *E[I]* using the centroid. Here,


$$L=\sqrt{{\left(Y_{1}\left(x\right)-\;y_{1}\left(x\right)\right)}^{2}+\;{\left(Y_{2}\left(x\right)-\;y_{2}\left(x\right)\right)}^{2}},$$

where *L* is the distance between the centroid *(Y_1_(x), Y_2_(x))* at the candidate data point, *x*, and the nearest point on the sub-optimal PF *(y_1_(x), y_2_(x))*. The centroid of the probability distribution for the candidate point in the beige-shaded region (electronic supplementary material, figure S8) is determined by evaluating the integral:


$$\int Y_{1}\left(x\right)=\;\int_{\text{shaded}}^{,}y_{1}\varphi \left(y_{1},y_{2}\right)dy_{2}dy_{1}/P\left[I\right].$$

For every candidate location in the region of improvement, the EI-Centroid is computed by multiplying P[I] with the smallest distance between points on the known sub-optimal PF and the centroid of the probability distribution within the zone of improvement. The candidate point with the highest EI-Centroid value is selected as the next measurement.

The Maximin approach [56] considers the average projected values for a candidate composition as ($\mu_{1},\mu_{2}$). We define the distance


$$L_{\text{maximin}}={\text{Max}}_{i}\left(\text{Min}\;(p_{i1}-\;\mu_{1},p_{i2}-\;\mu_{2}\right),$$

where $P_{i}=(p_{i1},p_{i2})$ and $P_{i}\in PF$. The Maximin *E[I]* is calculated as $E{\left[I\right]}_{\text{maximin}}=\;L_{\text{maximin}}\times P\left[I\left(x\right)\right]$, by multiplying P[I] with the maximum value obtained from the shortest distance between the means $\left(\mu_{1},\mu_{2}\right)$ of a specific candidate point and the individual sub-optimal PF points $p_{i}$.

The *E[I]*-Centroid method evaluates the combined improvement of properties $y_{1}$ and $y_{2}$, while the *E[I]*-Maximin method evaluates each property separately. It selects the smaller property value from a specific sub-optimal Pareto point and then maximizes it over all sub-optimal Pareto points. Both algorithms choose a data point that maximizes the modification of the sub-optimal PF through its measurement. We employed both the *E[I]*-Centroid and *E[I]*-Maximin strategies and conducted a comparative analysis against (i) random selection, (ii) pure exploitation using solely the mean values of predictions from an ML model and finally (iii) pure exploration, where the selection is determined by the magnitude of the variance for candidate points in the region of improvement. The design process we use is shown in electronic supplementary material, figure S1.

## 3. Results and discussion

### (a) Active learning for single-objective optimization of Invar and magnetic alloys

#### (i) Data distribution in the latent space of Invar and magnetic alloys

**Figure 2.**
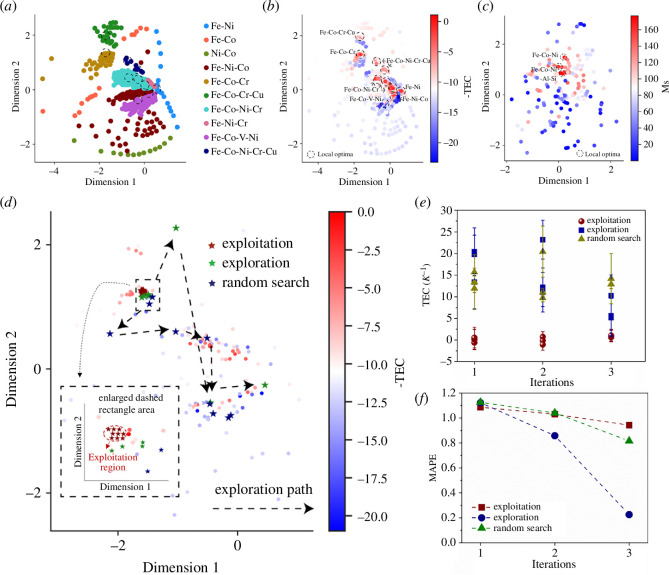
(*a*) WAE latent space of the alloys related to the Invar dataset. Different alloy systems are marked with different colours and tend to cluster separately. (*b*) and (*c*) the latent distribution of the alloys marked with different colours according to the magnitude of their TEC (Invar alloys) and M_S_ (magnetic alloys). The local optima regions in different alloy systems are marked with dashed cycles. (*d*) The latent distribution of the Invar alloys was sampled with different strategies, i.e. exploitation, exploration and random search. (*e*) and (*f*) the change of TEC and MAPE with different strategies, i.e. exploitation, exploration and random search.

WAE is used to visualize the distribution of the alloys in a latent space [26]. Figure 2*a* shows the latent distribution of the Invar alloys after dimensional reduction. In the latent space, distinct alloy systems are differentiated by varying colours, forming clusters in separated regions. Simultaneously, diverse alloy systems exhibit a regular distribution pattern. For instance, MEAs and HEAs appear disrupted towards the centre, while binary and ternary alloys tend to surround them. Figure 2*b,c* depicts the latent distribution of alloys, with colour-coding based on their TEC for Invar alloys and MS for magnetic alloys, respectively. Notably, dashed circles are used to highlight local optima within the latent space. In figure 2*b*, local optima regions are conspicuous for Invar alloys, notably within the Fe-Ni, Fe-Ni-Co, Fe-Co-Cr, Fe-Co-Ni-Cr, Fe-Co-V-Ni and Fe-Co-Ni-Cr-Cu alloy systems. Conversely, in figure 2*c*, representing magnetic alloys, the local optima are primarily observed within the Fe-Co-Ni and Fe-Co-Ni-Al Si systems.

We noted two key distinctions in the latent distributions of the datasets. First, the magnetic dataset contains about half the number of alloys compared to the Invar database. Second, fewer local optima are observed in the latent space of the magnetic dataset compared to the Invar database. These variations are typical across different material datasets, which is why we chose to illustrate with the Invar and magnetic datasets in this study. The variance in local optima across different material datasets arises from the complex composition–property relationships inherent in each material system and significantly impacts the balance between exploitation and exploration strategies, as discussed in §3a(iii).

#### (ii) Illustration of pure exploitation and exploration strategies of Invar alloys

In this section, we delve into a comprehensive understanding of exploitation and exploration strategies, focusing on insights drawn from the Invar dataset. We used the framework shown in figure 1. Three iterations were conducted using pure exploitation and exploration strategies, compared with random search. The outcomes are depicted in figure 2*d–f* and summarized in table 1. Figure 2*d* visualizes the latent space of the sampled alloys based on different strategies, denoted by distinct coloured pentagrams. The exploitation represented by red pentagrams predominantly selects alloys near the Fe-Co-Cr local optimum, exhibiting a reluctance to explore unknown regions. Conversely, both the exploration (green pentagrams) and random search (blue pentagrams) demonstrate a more diverse exploration path, venturing into potential local optima regions beyond the immediate vicinity. In figure 2*e* and table 1, compared to random search (olive triangles), the exploitation tends to sample alloys with very low TEC (TEC ≈ 0 K^−1^) (red balls), which fits the requirement of low-TEC alloy discovery. In contrast, the exploration (blue rectangles) tends to sample alloys with large uncertainties (more than two times larger than the uncertainties in the exploitation), aiming at exploring the unknown region. However, these alloys exhibit relatively high TEC (TEC > 5 K^–1^). Figure 2*f* shows the change of MAPE of the testing dataset in three iterations with different strategies. The exploitation, exploration and random search are marked with red rectangles, blue circles and olive triangles, respectively. Notably, the exploration demonstrates significantly greater decreases in error compared to the other two strategies. Moreover, the random search exhibits a slightly more pronounced decreasing trend than exploitation.

**Table 1.** Predicted mean and uncertainty of the alloys in three iterations with exploitation, exploration and random search strategies.

iteration	strategy	alloy	mean TEC (×10^−6^ K^−1^)	uncertainty (×10^−6^ K^−1^)
1	exploitation	1	1.19	1.55
		2	1.81	2.35
		3	1.86	2.39
	exploration	1	7.77	6.16
		2	22.60	5.51
		3	19.71	4.44
	random search	1	7.04	6.32
		2	15.28	0.91
		3	12.98	0.92
2	exploitation	1	1.22	1.19
		2	1.23	1.23
		3	1.24	1.31
	exploration	1	5.66	4.56
		2	15.66	4.49
		3	6.33	4.45
	random search	1	21.64	5.91
		2	10.71	0.90
		3	12.08	0.90
3	exploitation	1	0.63	0.85
		2	0.90	1.34
		3	1.04	1.42
	exploration	1	10.26	4.79
		2	5.61	4.60
		3	5.18	4.44
	random search	1	14.20	5.80
		2	12.88	1.03
		3	12.92	1.04

In summary, while the exploitation efficiently identifies target alloys, it is prone to getting trapped in local optima. Conversely, exploration and random search facilitate the exploration of unknown regions, mitigating the risk of being stuck in local optima. Moreover, they prove most effective in error reduction and enhancing the model’s performance.

#### (iii) The exploration and exploitation strategies balance of Invar and magnetic alloys

**Figure 3.**
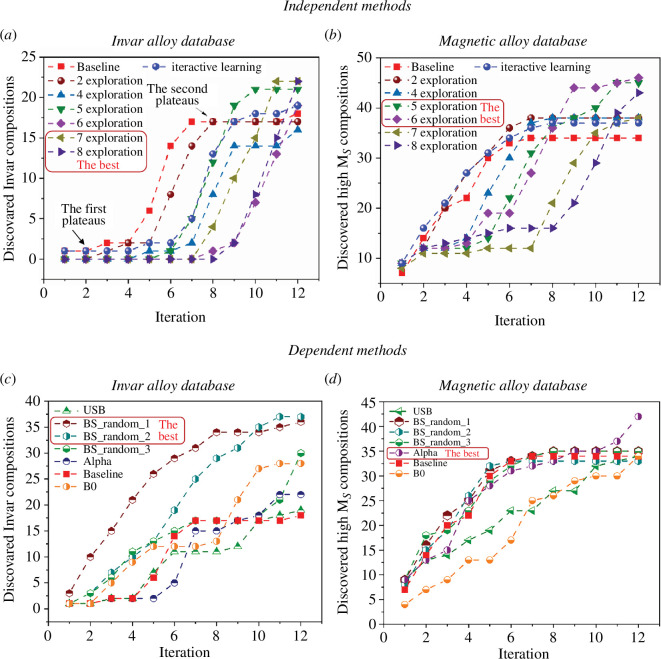
The number of discovered Invar and high magnetization compositions after 12 iterations with different exploitation and exploration strategies. (*a*) and (*b*) Independent methods. (*c*) and (*d*) Dependent methods. We show 3 BS results with different random seeds.

Balancing exploration and exploitation strategies aims to minimize the number of iterations required for discovering new materials. However, finding the optimal balance varies across databases and materials, posing a significant challenge. While existing research has explored this issue [34,56], there is no universally applicable strategy for all optimization problems. In this study, with the framework shown in figure 1, we employ dependent and independent methods alternatively to tackle this challenge, aiming to determine when to combine exploitation and exploration in one iteration or in the whole process. Detailed descriptions of these methods can be found in §2d, with corresponding results illustrated in figure 3.

In figure 3*a*, the discovery of Invar alloys using independent methods is depicted with the increase of active learning iterations. In the brute force search, we initially conduct various levels of exploration—0, 2, 4, 5, 6, 7 and 8 explorations—before transitioning to exploitation. The strategy with 0 exploration serves as the baseline, denoted by the red rectangles. The baseline curve experiences an initial plateau followed by a rapid increase phase. Around the seventh iteration, the curve begins to plateau again, suggesting potential entrapment in local optima. By gradually increasing the number of explorations at the outset, the curves progressively overcome this second plateau. Notably, employing 7 or 8 explorations results in a significantly higher number of discovered alloys after 12 iterations compared to the baseline, highlighting the critical role of exploration in the brute force search. We also show the results of interactive learning in figure 3*a*, which is indicated in blue spheres. The results achieved through interactive learning surpass those of the baseline.

In figure 3*b*, the number of discovered compositions with high *M*s (*M*s > 120 Am^2^ kg^−1^) after 12 iterations is depicted. Unlike the Invar database, all strategies in the independent methods outperform the baseline (pure exploitation). Optimal performance is achieved with 5–6 explorations, contrasting with the Invar dataset, where 7–8 explorations yield the best results. We speculate that database size and the number of local optima influence the most effective methods. In datasets with fewer data and local optima (magnetic alloy dataset), independent strategies with relatively fewer explorations prove advantageous.

In figure 3*c–d*, we depict the results obtained with dependent methods, including the UCB, BS, Alpha methods and BO. Three BS results with different random seeds are shown to account for sampling randomness, with similar outcomes observed across different seeds. Further insights into the effect of random seeds are provided in electronic supplementary material, figure S5. The same baseline, as shown in figure 3*a*, is included for comparison. In figure 3*c*, illustrating results from the Invar dataset, all dependent methods exhibit superior performance to the baseline, with BS yielding the best results. Conversely, in figure 3*d*, representing the magnetic dataset results, the Alpha method demonstrates the most favourable performance. We speculate that this discrepancy arises from BS prioritizing exploration while Alpha emphasizes exploitation.

Overall, both independent and dependent methods have proven more effective than pure exploitation, which serves as the baseline. However, their performance varies depending on specific scenarios. In situations where there is a substantial amount of data and a high number of local optima, such as in the Invar dataset, these strategies demonstrate greater advantages when they emphasize exploration, such as brute force search with 7–8 explorations. Conversely, in scenarios where data are relatively scarce and the number of local optima is limited, as observed in the magnetic dataset, these strategies excel when emphasizing exploitation, such as Alpha methods.

When comparing independent and dependent methods for the same alloy system, interesting discoveries emerge. For Invar alloys, dependent methods outperformed independent methods, while for magnetic alloys, the opposite was observed. This discrepancy is due to the substantial amount of data and high number of local optima in the Invar alloys dataset, necessitating more explorations. Dependent methods guarantee exploration in each iteration, whereas independent methods may miss exploration to some extent.

Comparing different methods, such as UCB, BS, Alpha and BO, presents several challenges: (i) the performance of each method can vary widely across datasets, making it difficult to consistently determine which is most accurate; (ii) even within the same method, results can be unstable due to factors like different random seeds in BS or varying kernel functions in GPR for BO; and (iii) the number of iterations also influences outcomes, as some methods excel in fewer iterations but may underperform with more.

However, it is important to mitigate the drawbacks of certain methods. For instance, independent methods are prone to falling into local optima—evident in the plateaus seen in figure 3*a,b*—because they focus solely on either exploitation or exploration in each iteration. In contrast, dependent methods can better avoid local optima by combining exploitation and exploration in each iteration.

For the UCB method, performance hinges on the model’s accuracy since the highest upper bound guides the selection of the next composition. The BS and Alpha methods adjust exploration and exploitation through specific parameters (*ρ* for BS and α for the Alpha method). The main challenge with these methods lies in determining the optimal parameter settings. BO, often utilizing GPR as a surrogate model, struggles with high-dimensional optimization problems, so caution is advised when dealing with large input dimensions.

In summary, conducting a closed-loop test can offer valuable insights into the most efficient optimization approach before integrating these surrogate models with experimental synthesis and characterization processes.

In the supplemental material, we address three factors influencing the balance between exploitation and exploration strategies. First, the number of newly sampled promising alloy compositions in each iteration plays a crucial role. Exploration becomes effective when sampling a large number of new compositions per iteration. Second, the impact of known data, or labelled data, is significant. In active learning for diverse materials, the unknown data pool is often infinite, while the known data pool depends on the dataset size. Results indicate a greater need for exploration with relatively smaller original datasets. Finally, the type of error in surrogate models affects performance. Variations in error types across classification or regression problems are observed, with certain choices, such as MAPE in Invar alloy design, enhancing the active learning process. Further discussion on these factors is provided in the supplemental material.

### (b) Active learning strategies for multi-objective optimization for steel dataset

**Figure 4.**
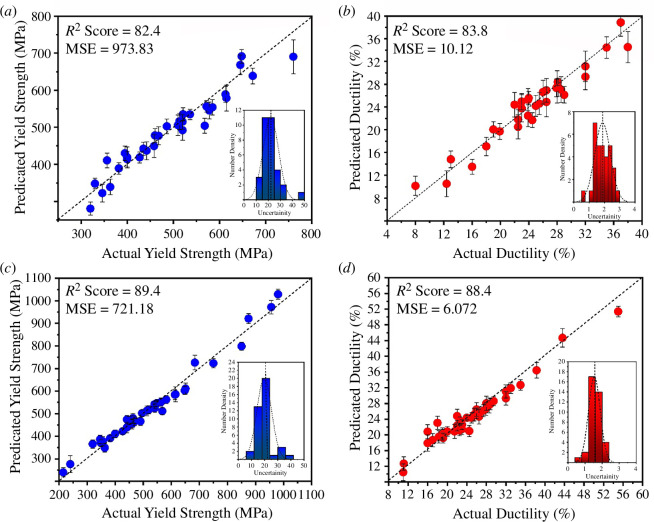
Performance of the surrogate RF model on the test dataset for (*a*) yield strength, (*b*) ductility for the initial dataset. Inset also shows the distribution of the uncertainties associated with the predictions. (*c*) and (*d*) show the performance of the RF model for the yield strength and ductility for the final dataspace after optimization.

Figure 4 illustrates the performance evaluation of the surrogate RF models (details of the RF models are provided in §2c) on both the initial testing dataset, depicted in figure 4*a,b*, and the final optimized composition space, depicted in figure 4*c,d*. In these figures, the effectiveness of the RF regressors in predicting yield strength and ductility is illustrated. Additionally, the spread of uncertainties associated with the predictions is visualized in the insets. The performance of the RF regressors has notably improved after the optimization of the composition space. This improvement is observed not only in terms of enhanced average performance but also in the significant reduction of uncertainty distribution associated with the predictions. Such a relatively narrow uncertainty distribution suggests that further refinement through pure exploitation or exploration strategies may yield limited or negligible improvements. This indicates a convergence towards an optimal solution within the current design framework.

The optimization of the composition space based on objective-driven criteria has played a pivotal role in enhancing the performance of the RF models within the active learning framework. Notably, the final test results underscore the transformative impact of this optimization, showcasing an augmentation in both yield strength and ductility. Whereas the initial compositional space constrained yield strength and ductility to 800 MPa and 40%, respectively, the optimized space facilitated the design of new compositions with elevated properties, reaching 1000 MPa and 60% for yield strength and ductility, respectively. This compelling outcome underscores the efficacy of optimization strategies in the discovery of novel TRIP steels characterized by improved strength and ductility trade-offs.

**Figure 5.**
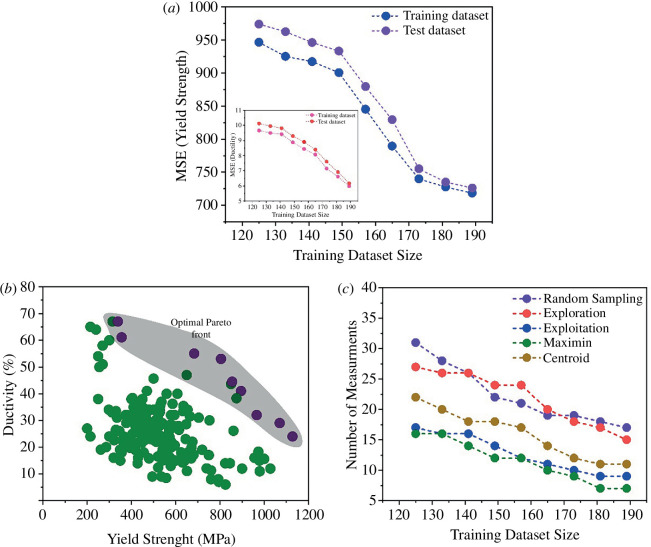
(*a*) The trend of MSE values for yield strength as the optimization continues and experimental feedback is provided to the RF model (inset shows the MSE trend for ductility); (*b*) the final optimal PF with optimized new compositions marked in purple in the grey shaded area; (*c*) the size of the training dataset is plotted against the average number of design cycles required to find all the points in optimal PF. The Maximin-based design strategy in which the exploration and exploitation of data are more balanced performs much better than all other methods.

Figure 5*a* provides a comprehensive depiction of the performance enhancement observed in surrogate RF regressors as the size of the training dataset increases, employing the Maximin method. The effect of the dataset has been discussed in the electronic supplementary material section. While conventional wisdom dictates that larger training datasets generally bolster the performance of ML models, it is noteworthy that the judicious addition of subsequent data points via optimization strategies yields substantial performance improvements. This is evidenced by the discernible trend of decreasing MSE values observed for both yield strength and ductility regressors. Notably, the utilization of the Maximin method during compositional optimization serves to expedite the selection of the next potential compositions, leveraging insights gleaned from existing compositions within the dataset. Consequently, this approach fosters accelerated optimization trajectories compared to conventional methods such as random selection, exploitation or pure exploration strategies.

In figure 5*b*, the optimal PF of the TRIP steel dataset, characterized by seven discernible points, is presented. This representation underscores the richness and complexity of the design space explored, which encompasses diverse training datasets ranging in the size from 125 to 196, collated from preceding iterations of optimization. Figure 5*c* demonstrates that employing multi-objective optimization design strategies decreases the required number of measurements to identify the optimal PF by approximately 30% compared to random selection. The Centroid-based design method and pure exploration yield similar results. The Maximin-based design strategy for materials discovery is more efficient than the Centroid-based strategy, pure exploitation or exploration to identify materials on the PF in the fewest iterations feasible. The performance also indicates that the pure exploration strategy is less efficient than random selection. This is because, in the context of pure exploration, the material with the highest uncertainty level is chosen for the subsequent experiment. This indicates that pure exploration entirely ignores the anticipated values of attributes and forces the design strategy to select a material that differs most from the existing data. The performance of pure exploitation is equal to that of the Centroid-based design. In Centroid-based design, the balance between exploration and exploitation is skewed towards exploration when the Centroid is far from the mean.

To explain the different behaviour of the various approaches, it is essential to consider the scale of the data associated with each of the properties: while the probability of improvement (*P[I]*) is a dimensionless measure that remains unaffected by the magnitude of the objective values, the *EI* measure quantifies the improvement and possesses dimensions. The *EI* prioritizes the goal attribute with larger magnitudes. To mitigate this bias, the property values of established datasets are standardized before each design iteration. Within the informatics-based design approach, we focus on situations with insufficient data. This leads to regression models that initially have low prediction accuracy and large levels of uncertainty, primarily because of significant cross-validation failures. In the Maximin-based method, the exploration component of the design process is governed by the probability of improvement *P[I]*, while the ‘distance’ *L*, which is impacted by the means, introduces the notion of exploitation. The technique demonstrated robust performance even when the initial accuracy of the RF regression model was inadequate to be considered dependable for making predictions. Nevertheless, the most optimal Pareto locations can be discovered within a limited number of design iterations. This highlights a feature of the design that is increasingly apparent in different materials challenges and datasets: the design is highly accommodating of an initial substandard surrogate model.

## 4. Conclusions

In this article, we elucidate the active learning strategies for both single- and multi-objective optimization. The main conclusions are: (i) we identified distinct roles of exploitation and exploration strategies within the active learning process. The exploitation strategy is efficient at finding the target alloys but easy to be trapped in the local optima. On the other hand, the exploration strategy can explore unknown regions to avoid getting stuck in local optima. (ii) For the single-objective optimization, in situations where there is a substantial amount of data and a high number of local optima (as observed in the Invar dataset), these strategies prove more advantageous when they emphasize exploration. Conversely, in scenarios characterized by a smaller amount of data and fewer local optima (as evident in the magnetic dataset), strategies become more advantageous when they lean towards relatively more exploitation. (iii) For multi-objective optimization, the Maximin-based method excels in robustness, especially when the initial accuracy of the RF regression model is insufficient for reliable predictions. This underscores a notable feature of the design, one that is increasingly evident in various materials challenges and datasets: the design is highly adaptable even when dealing with an initially substandard surrogate model.

## Data Availability

The source codes of the ML models, together with the data, are deposited onto the repository of Github [57] for free download. Supplementary material is available online [58].
